# Sarcopenia negatively affects hip structure analysis variables in a group of Lebanese postmenopausal women

**DOI:** 10.1186/s12859-020-3353-9

**Published:** 2020-03-11

**Authors:** Hayman Saddik, Riad Nasr, Antonio Pinti, Eric Watelain, Ibrahim Fayad, Rafic Baddoura, Abdel-Jalil Berro, Nathalie Al Rassy, Eric Lespessailles, Hechmi Toumi, Rawad El Hage

**Affiliations:** 1 Department of Physical Education, University of Balamand, Kelhat, El-Koura Lebanon; 2 I3MTO, EA4708, Université d’Orléans, Orléans, France; 3 DeVisu - Design, Visuel, Urbain, EA 2445, UVHC, Valenciennes, France; 4 Laboratoire Impact de l’Activité Physique sur la Santé (IAPS), Université de Toulon CS 60584- 83041 TOULON CEDEX 9, Toulon, France; 5 Department of Rheumatology, Hôtel-Dieu Hospital, Saint Joseph University, Beirut, Lebanon; 6 6EA-3300, APERE, University of Picardie Jules Verne, Amiens, France; 7 Laboratory of Pathophysiology of Inflammatory Bone Diseases PMOI EA4490, University of Littoral Opal Coast ULCO, Boulogne sur Mer, France; 8 University of Lille, Lille, France

**Keywords:** Sarcopenia, DXA imaging, Bone strength indices, Fracture risk, Menopause

## Abstract

**Background:**

The current study’s purpose is to compare hip structural analysis variables in a group of postmenopausal women with sarcopenia and another group of postmenopausal women with normal skeletal muscle mass index. To do so, the current study included 8 postmenopausal women (whose ages ranged between 65 and 84 years) with sarcopenia and 60 age-matched controls (with normal skeletal muscle mass index (SMI)). Body composition and bone parameters were evaluated by dual-energy X-ray absorptiometry (DXA).

**Results:**

Weight, lean mass, body mass index, femoral neck cross-sectional area (FN CSA), FN section modulus (Z), FN cross sectional moment of inertia (CSMI), intertrochanteric (IT) CSA, IT Z, IT CSMI, IT cortical thickness (CT), femoral shaft (FS) CSA, FS Z and FS CSMI were significantly greater (*p* < 0.05) in women with normal SMI compared to women with sarcopenia. In the whole population, SMI was positively associated with IT CSA, IT Z, IT CSMI, IT CT, FS CSA, FS Z, FS CSMI, FS CT but negatively correlated to IT buckling ratio (BR) and FS BR.

**Conclusion:**

The current study suggests that sarcopenia has a negative effect on hip bone strength indices in postmenopausal women.

## Background

In elderly humans, muscle mass decreases with age leading to “sarcopenia” or decreased muscle mass [1, 2]. The life span of human beings has been steadily increasing in the past decades, and this has led to sarcopenia becoming more common; therefore, the impact of this disease on society is becoming more prevalent [3]. Lean mass is a major predictor of bone mineral density (BMD) and geometric indices of hip bone strength in elderly subjects [4–9]. In this population as well, fat mass is another determinant of BMD and geometric indices of hip bone strength [4–9]. Several studies have previously elucidated the mechanisms explaining the link between fat mass and bone parameters [4–9]. Lately, sarcopenia has been associated with low BMD values in the elderly [10, 11]. However, bone strength is influenced by both BMD and bone geometry [12–14]. Several studies have shown that hip structure analysis (HSA) variables can predict incident hip fracture risk in postmenopausal women [15–23]. In line with this, we have recently shown that sarcopenia has a negative effect on hip bone strength indices in elderly men [24]. On the contrary, in elderly women, the relationship between sarcopenia and hip bone strength indices has not been completely elucidated. The current study’s purpose was to compare HSA variables in postmenopausal women with sarcopenia and postmenopausal women with normal skeletal muscle mass index.

## Methods

### Subjects and study design

This study included 8 postmenopausal women (aged between 65 and 84 years; 71.6 ± 4.7 years) with sarcopenia and 60 age-matched (aged between 65 and 84 years; 75.3 ± 6.6 years) controls (with normal skeletal muscle mass index). The women were randomly chosen from the greater Beirut area, Lebanon.

### Exclusion criteria

The subjects that were excluded from the study were those suffering from any medical condition which could potentially affect bone metabolism such as history of chronic disease with vital organ involvement or intake of medications that may affect bone metabolism (i.e., steroid intake for more than 6 months and/or treatment with bone antiresorptive drugs). In addition, other subjects that were excluded were those with a radiotherapy or chemotherapy history or those who had been in bed rest for more than 1 month 6 months prior to the study. Other excluded subjects were those with conditions that technically interfere with dual-energy X-ray absorptiometry (DXA) assessment (i.e., previous spine or hip surgery). The Institutional Review Board of Hotel-Dieu Hospital, Saint Joseph University approved of this study, and all the subjects participating in it provided informed consent.

### Anthropometric measurements

The subjects stood in an upright position for their height (in centimeters) to be measured to the nearest 1 mm with a Seca standard stadiometer (SecaGmbH, Hamburg, Germany). As for body weight, it was measured in kilograms on a Taurus mechanic scale with a precision of 100 g. The women wore underclothes solely while being weighed. BMI was calculated as body weight divided by height squared (kilogram per square meter). Body composition was evaluated by DXA (HologicQDR-4500 W; Hologic Inc., Waltham, MA). In our medical center, the in vivo coefficients of variation were < 1% for fat and lean mass [4, 5].We used the skeletal muscle mass index (appendicular skeletal mass (ASM)/height^2^) to define sarcopenia as previously reported [1]. Based on DXA results, we calculated the ASM for each participant as the sum of the upper and lower limb muscle mass without fat and bone tissue. A skeletal muscle mass index (SMI) < 5.5 kg/m^2^ for women was defined as the cut-off point for sarcopenia [1]. The European Working Group on Sarcopenia in Older People to define sarcopenia in women determined this cut-off point [2].

### Measurements of bone variables

Dual-energy X-ray absorptiometry (Hologic QDR-4500 W, Waltham, MA USA) measurements were taken to evaluate bone mineral content (BMC) and bone mineral density (BMD). Both bone parameters were determined for each individual by DXA at whole body, lumbar spine and proximal femur (total hip [TH] and FN). In our laboratory, the coefficients of variation were < 1% for BMC and BMD [25–28]. All analyses were performed by the same certified technician who used the exact same technique for all measurements. The Hip Structure Analysis (HSA) program was used to assess hip strength indices by analyzing DXA scans at the femoral neck (FN), the intertrochanteric region (IT), and the femoral shaft (FS). The technical aspect of the HSA program was precisely explained in two previous studies [11, 12]. In this study, we analyzed three regions of the hip: FN at its narrowest region, the IT region, and the FS. Bone CSA (in square centimeter) and Z (in cubic centimeter) were measured at the 3 regions as previously described [11, 12]. Cross sectional area (CSA), which is an index of axial compression strength, section modulus (Z), which is an index of bending strength, cross sectional moment of inertia (CSMI), which is an index of structural rigidity, cortical thickness (CT), which is an estimate of mean cortical thickness, and buckling ratio (BR), which is an index of bone geometric instability, were measured by the HSA program [4, 12, 29]. Mechanically, CSA is a resistance indicator to loads directed along the bone axis. Z is a strength indicator of the bone which shows to what extent the bone can resist bending and torsion [29]. In this study, we also calculated BR, which is an index of susceptibility to local cortical buckling under compressive loads [29]. The buckling ratio is considered an estimate of relative cortical thickness (subperiosteal radius/cortical thickness). Higher Buckling Ratio values mean greater instability, and they are associated with increased fracture risk [30–33]. All HSA analyses were completed by one certified technician. In our medical center, the coefficients of variation for CSA and Z at the 3 regions (FN, IT, and FS) assessed by duplicate measurements in 10 women are < 3%.

### Statistical analysis

All clinical data and bone measurements had their standard deviations and means calculated. Gaussian distribution was checked when comparing the two groups (sarcopenic women and women with normal SMI). Parametric unpaired t-tests were used when Gaussian distribution was found. Mann-Whitney U tests were used in other cases. Associations between clinical characteristics and DXA parameters were given as Pearson correlation coefficients. HSA variables were compared between the two groups (sarcopenic women and women with normal SMI) after adjustment for lean mass and body weight using a one-way analysis of covariance (ANCOVA). Data are analyzed with Number Cruncher Statistical System (NCSS, 2001). A level of significance of *p* < 0.05 was used.

## Results

### Clinical characteristics and bone measurements of the study population

Weight, body mass index, lean mass, fat mass, FN CSA, FN Z, FN CSMI, IT CSA, IT Z, IT CSMI, IT CT, FS CSA, FS Z and FS CSMI were significantly higher (*p* < 0.05) in women with normal SMI compared to women with sarcopenia (Table 1). IT BR was significantly higher (p < 0.05) in women with sarcopenia compared to women with normal SMI.

**Table 1:** Clinical characteristics and bone variables in sarcopenic women and normal women

	Women with normal SMI (*n* = 60)	Sarcopenic women (*n* = 8)	*P*-value
Age (years)	71.633 ± 4.780	75.375 ± 6.610	0.051
Weight (kg)	70.233 ± 12.500**	55.875 ± 9.141	0.003
Height (m)	1.519 ± 0.0596	1.504 ± 0.0752	0.503
BMI (kg/m^2^)	30.343 ± 4.745**	24.653 ± 3.059	0.002
FM (kg)	27.520 ± 7.810*	20.578 ± 6.490	0.019
LM (kg)	39.986 ± 5.344***	31.236 ± 2.827	< 0.001
SMI (kg/m^2^)	7.22 ± 1.40	5.31 ± 0.19	< 0.001
WB BMD (g/cm^2^)	0.884 ± 0.0841	0.834 ± 0.0618	0.106
WB BMC (kg)	1.528 ± 0.280*	1.285 ± 0.202	0.021
L1-L4 BMD (g/cm^2^)	0.783 ± 0.158	0.746 ± 0.0821	0.827
TH BMD (g/cm^2^)	0.727 ± 0.124*	0.616 ± 0.0894	0.017
FN BMD (g/cm^2^)	0.632 ± 0.100**	0.532 ± 0.0871	0.009
FN CSA (cm^2^)	2.214 ± 0.405**	1.821 ± 0.212	0.009
FN CSMI (cm^2^)^2^	1.993 ± 0.656*	1.408 ± 0.261	0.01
FN Z (cm^3^)	1.072 ± 0.284*	0.801 ± 0.159	0.01
FN CT (cm)	0.140 ± 0.034	0.118 ± 0.026	0.08
FN BR	13.92 ± 2.94	16.28 ± 6.75	0.08
IT CSA (cm^2^)	3.726 ± 0.836**	2.882 ± 0.356	0.007
IT CSMI (cm^2^)^2^	9.36 ± 3.05*	6.53 ± 1.25	0.01
IT Z (cm^3^)	3.090 ± 0.879**	2.208 ± 0.399	0.003
IT CT (cm)	0.287 ± 0.066*	0.231 ± 0.034	0.02
IT BR	11.1 ± 2.7 *	13.1 ± 2.6	0.04
FS CSA (cm^2^)	3.520 ± 0.647**	2.853 ± 0.343	0.006
FS CSMI (cm^2^)^2^	3.330 ± 0.774***	2.350 ± 0.363	< 0.001
FS Z (cm^3^)	2.094 ± 0.419***	1.516 ± 0.237	< 0.001
FS CT (cm)	0.429 ± 0.098	0.360 ± 0.068	0.05
FS BR	3.91 ± 1.16	4.53 ± 1.34	0.16

*BMI* Body Mass Index, *SMI* Skeletal Muscle Mass Index, *WB BMC* Whole Body Bone Mineral Content, *WB BMD* Whole Body Bone Mineral Density, *TH* Total hip, *FN* Femoral Neck, *FN CSA* Femoral Neck Cross-Sectional Area, *CSMI* cross sectional moment of inertia, *Z* Section modulus, *CT* Cortical Thickness, *IT* Intertrochanteric, *FS* Femoral shaft, *BR* Buckling Ratio; * *p* < 0.05; ** *p* < 0.01; *** *p* < 0.001

### Associations between body weight, lean mass, fat mass, SMI and hip structure analysis variables

**Fig. 1:**
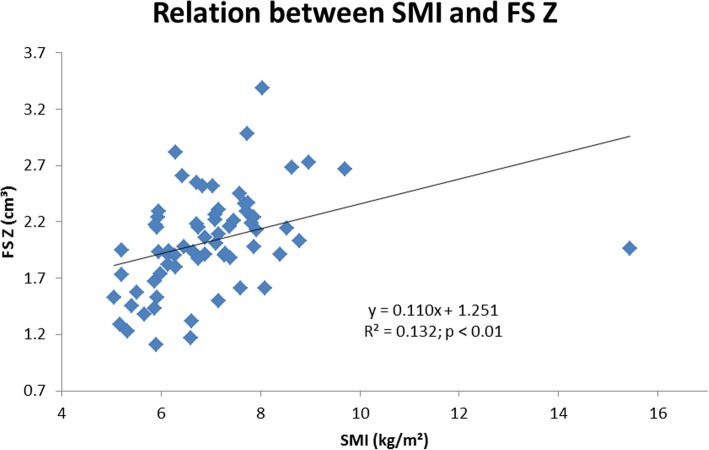
Relation between skeletal muscle mass index and femoral shaft section modulus

**Fig. 2:**
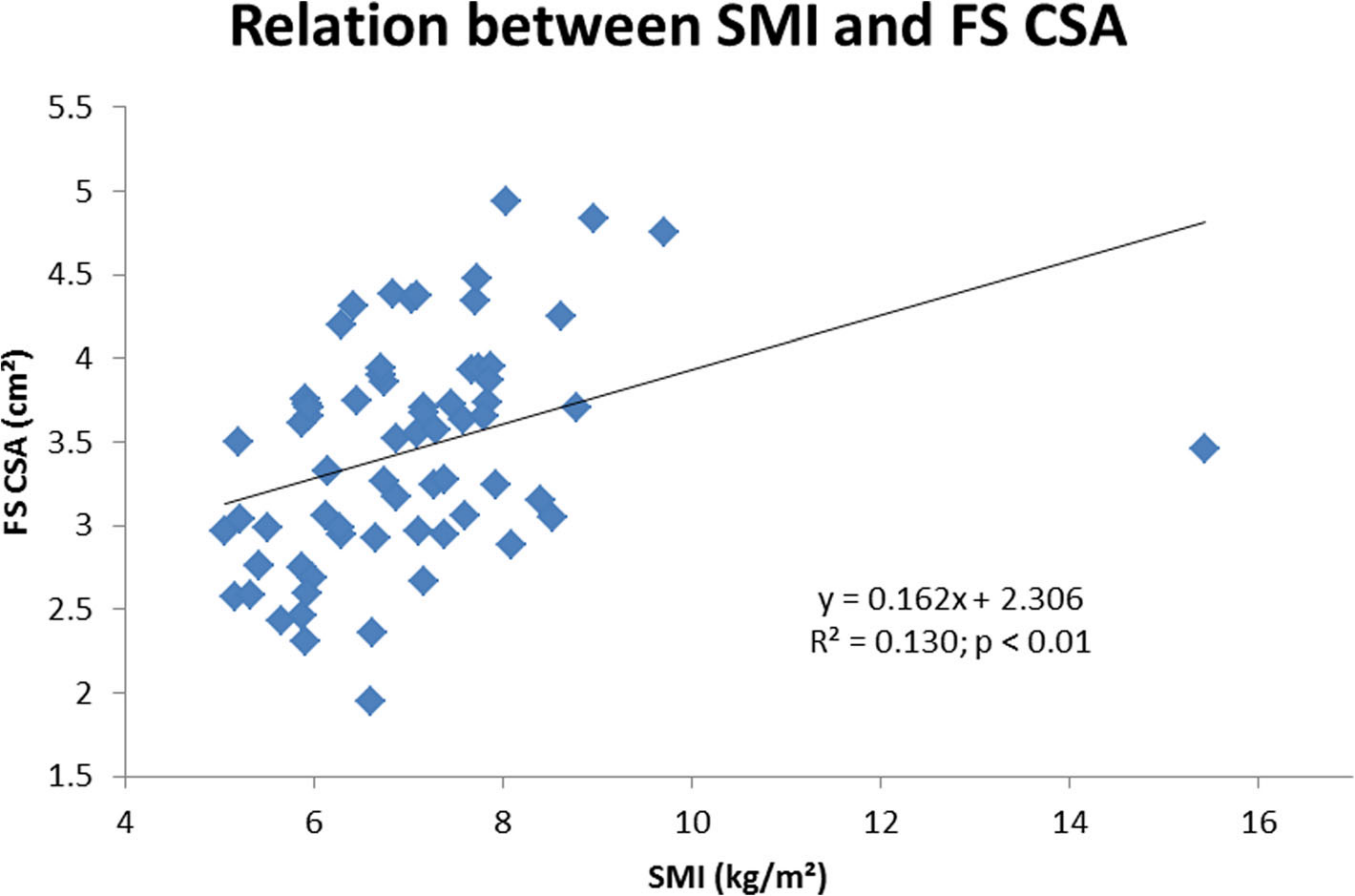
Relation between skeletal muscle mass index and femoral shaft cross-sectional area

In the whole population (Table 2), body weight, lean mass and fat mass were positively correlated with CSA, CSMI, and CT at the FN, IT, and FS (*p* < 0.01) and negatively correlated with BR at the IT and FS (*p* < 0.01). SMI (kg/m^2^) was positively correlated to IT CSA (r = 0.30; *p* < 0.05), IT Z (r = 0.28; p < 0.05), IT CSMI (r = 0.24; p < 0.05), IT CT (r = 0.29; p < 0.05), FS CSA (r = 0.36; p < 0.01), FS Z (r = 0.36; p < 0.01), FS CSMI (r = 0.32; p < 0.01), FS CT (r = 0.32; p < 0.01) but negatively correlated to IT BR (r = − 0.31; p < 0.05) and FS BR (r = − 0.23; p < 0.05) (Figs. 1 and 2).

**Table 2:** Correlations between body weight, lean mass, SMI and bone variables in the whole population

	Body weight (kg)	Lean mass (kg)	Fat mass (kg)	SMI (kg/m^2^)
WB BMC (g)	0.720**	0.581**	0.673**	0.295*
WB BMD (g/cm^2^)	0.511**	0.412**	0.461**	0.267*
L1-L4 BMD (g/cm^2^)	0.282*	0.243*	0.276*	0.090
TH BMD (g/cm^2^)	0.649**	0.503**	0.663**	0.323**
FN BMD (g/cm^2^)	0.533**	0.455**	0.505**	0.258*
FN CSA (cm^2^)	0.512**	0.519**	0.449**	0.218
FN CSMI (cm^2^)^2^	0.359**	0.435**	0.280*	0.193
FN Z (cm^3^)	0.379**	0.423**	0.320**	0.182
FN CT (cm)	0.452**	0.405**	0.411**	0.228
FN BR	−0.227	−0.104	−0.271*	−0.045
IT CSA (cm^2^)	0.641**	0.600**	0.589**	0.308*
IT CSMI (cm^2^)^2^	0.596**	0.623**	0.504**	0.242*
IT Z (cm^3^)	0.609**	0.631**	0.511**	0.287*
IT CT (cm)	0.595**	0.502**	0.574**	0.299*
IT BR	−0.504**	−0.380**	−0.508**	−0.309*
FS CSA (cm^2^)	0.689**	0.635**	0.664**	0.361**
FS CSMI (cm^2^)^2^	0.642**	0.716**	0.555**	0.322**
FS Z (cm^3^)	0.688**	0.717**	0.626**	0.364**
FS CT (cm)	0.590**	0.479**	0.599**	0.329**
FS BR	−0.454**	−0.288*	−0.516**	−0.253*

*FN* Femoral Neck, *TH* Total Hip, *WB BMC* Whole Body Bone Mineral Content, *WB BMD* Whole Body Bone Mineral Density, *FN CSA* Femoral Neck Cross-Sectional Area, *CSMI* cross sectional moment of inertia, *Z* Section modulus, *CT* Cortical Thickness, *BR* Buckling Ratio, *IT* Intertrochanteric, *FS* Femoral Shaft; * *p* < 0.05; ** *p* < 0.01

### DXA adjusted variables

After adjusting for lean mass using ANCOVA, no significant differences were observed between the two groups regarding all DXA parameters (WB BMC, WBBMD, L1-L4 BMD, TH BMD, FN BMD, FN CSA, FN CSMI, FN Z, IT CSA, IT CSMI, IT Z, IT CT, FS CSA, FS CSMI and FS Z).

After adjusting for body weight using ANCOVA, no significant differences were observed between the two groups regarding several DXA parameters (WB BMC, WBBMD, L1-L4 BMD, TH BMD, FN BMD, FN CSA, FN CSMI, FN Z, IT CSA, IT CSMI, IT Z, IT CT, FS CSA and FS CSMI) except for the FS Z which remained significantly higher in non-sarcopenic women compared to sarcopenic women.

## Discussion

This study which was conducted on a group of Lebanese post-menopausal women mainly demonstrates that sarcopenia is associated with lower geometric indices of hip bone strength. Moreover, the differences between the two groups (sarcopenic and non-sarcopenic women) regarding hip geometry indices disappeared after adjusting for lean mass. Thus, this study suggests that hip bone strength indices are adapted to lean mass in postmenopausal-women.

Weight, BMI, lean mass and fat mass were significantly higher in non-sarcopenic women compared to sarcopenic women. Sarcopenia is associated with significant changes in body weight and body composition which affect bone variables [34].

Lean mass and SMI were positive determinants of bone strength indices in our study. This result is in accordance with those of several studies conducted in elderly subjects [24, 35–38]. Higher SMI values correspond to higher dynamic loads imposed on bones [35–38]. These types of loads, known to activate an adaptive response, increase bone strength and decrease bone loss at an advanced age [35–38].

In our current study, the strongest determinants of bone variables were body weight and fat mass. Our results are in line with those of Reid et al. [39, 40] who demonstrated that fat mass is among the strongest determinants of BMD in menopausal women. In fact, fat mass may affect BMD by several mechanisms. The first mechanism is when increased fat mass raises mechanical loading on the skeleton [41]. Second, increased fat mass is associated with higher insulin, leptin, amylin and preptin circulating levels and lower adiponectin circulating levels [42]. Insulin, leptin, amylin and preptin have peripheral osteogenic effects via the stimulation of osteoblasts or the inhibition of osteoclasts while adiponectin enhances the osteoblast production of RANKL and constrains the production of osteoprotegerin (OPG) and is negatively correlated to BMD [43]. Third, free estrogen levels are increased in overweight and obese women [44, 45]. Fourth, another characteristic of fat mass is its ability to absorb environmental toxins thereby acting as a protector of other tissues in the body from the hazardous effects of those toxins [46–48]. Therefore, the larger the fat mass is, the lower the circulation of those environmental toxins will be; this will result in a decrease of the negative impact of these toxins on bone mass during the important bone formation years [46–48]. To sum up, age, gender and exercise status seem to influence the relationship between fat mass and bone mass [5, 9, 49–51]. For instance, fat mass excess is a risk factor for fracture in pre-pubertal children but protects against fracture in elderly [52].

In our study, sarcopenic women displayed lower hip BMD values compared to non-sarcopenic women. In addition, FN CSA, FN Z, FN CSMI, IT CSA, IT Z, IT CSMI, IT CT, FS CSA, FS Z and FS CSMI were significantly higher in women with normal SMI compared to women with sarcopenia while IT BR was significantly higher in women with sarcopenia compared to women with normal SMI. Higher buckling ratio values indicate greater cortical instability and thus increased risk of hip fracture [23, 29]. These results suggest that sarcopenia is associated with lower bone strength at the hip in menopausal women. All these differences disappeared after adjusting for lean mass and most of these differences disappeared after adjusting for body weight. Thus, this study suggests that hip bone strength indices adapt properly to body weight and lean mass in menopausal women.

There were some limitations in the current study. First, the cross-sectional nature of the study was the reason for not having a good assessment of a causal mechanical relationship between SMI and hip strength variables. Second, our small sample size may be the reason behind the lack of statistical significance for some variables. Third, we did not assess endocrine factors which are well-known to have an impact on BMD in elderly women such as growth hormone, insulin-like growth factor 1, testosterone, estrogen, sex hormone-binding globulin and dehydroepiandrosterone. Fourth, we also did not evaluate other predictors of BMD and HSA indices like physical activity level, daily protein intake, daily calcium intake and vitamin D status. Finally, DXA cannot make a distinction between subcutaneous and visceral fat, or between subcutaneous and intramuscular peripheral fat; however, subcutaneous and visceral fat may have different effects on bone geometry and strength [53, 54]. Nevertheless, up to our knowledge, the current study is one among few others that has explored the effect of sarcopenia on hip strength indices in elderly women in the Middle-East region. Our results are in accordance with those of three recent studies that aimed at studying the relationships between sarcopenia and bone strength parameters [55–57].

## Conclusions

This current study suggests that sarcopenia can negatively affect geometric indices of hip bone strength in postmenopausal women. Therefore, in order to prevent osteoporosis in postmenopausal women, implementing strategies to increase SMI is advised.

## Data Availability

The datasets used and/or analyzed during the current study are available from the corresponding author on reasonable request.

## Abbreviations

ALM: Appendicular Lean Mass
ASM: Appendicular Skeletal Muscle Mass
BMC: Bone Mineral Content
BMD: Bone Mineral Density
BMI: Body Mass Index
BR: Buckling Ratio
CSA: Cross-Sectional Area
CSMI: Cross Sectional Moment of Inertia
CT: Cortical Thickness
FN: Femoral Neck
FS: Femoral Shaft
IT: Intertrochanteric
SMI: Skeletal Muscle Mass Index
TH: Total Hip
WB: Whole Body
Z: Section Modulus
